# Austerity, Short-term Economic Recovery and Public Perception of Immigration in Ireland

**DOI:** 10.1007/s12115-022-00712-0

**Published:** 2022-04-26

**Authors:** Mathew Creighton, Egle Gusciute, Frances McGinnity

**Affiliations:** 1 School of Sociology, UCD Geary Institute for Public Policy, University College Dublin, Dublin, Ireland; 2 School of Sociology, University College Dublin, Dublin, Ireland; 3 Economic and Social Research Institute (ESRI), Dublin, Ireland

**Keywords:** Migration, Public opinion, Financial crisis, Austerity

## Abstract

The economic crisis of 2007/2008 did not affect all members of the European Union (EU) to the same extent. In the Irish case, the economic crisis and subsequent period of austerity paralleled an erosion in public support for immigration. However, little is known about how public perception changed during a period of short-term economic recovery, like that experienced in Ireland from 2014 to 2018. Using repeated cross-sectional survey data unique to Ireland, this work captures change in attitudes towards immigrants during the pre-crisis and late-austerity periods. Moreover, this research evaluates the importance placed on two immigrant attributes intimately linked to the labour market — education and skills. We provide evidence of an emergence of more moderate views of immigration during the recovery period, but only in the perceived importance of educational qualifications. Perception of skills remains notably unchanged. Of note, both attributes remain more important in the public eye relative to before the economic crisis. In other words, short-term economic recovery does not automatically translate into a more welcoming reception. We confirm that crises and periods of austerity erode public perception of newcomers, particularly when immigration is framed in terms of skill-based economic contribution. However, this work reveals some of the scars of a rapid and deep economic downturn alter the context of reception in a durable way, which remains notably resistant to short-term recovery.

In the European Union (EU), the financial crisis of 2007/2008 was unevenly experienced by member states, and Ireland — along with Spain, Portugal and Greece — was one of the countries most affected. The aftermath of the downturn coupled restricted national budgets with a rapid rise in unemployment (Roche et al. 2017; Ó Riain 2014). Austerity measures, in particular, reduced social supports and public sector wage cuts were protracted. In the case of Ireland, unemployment remained above pre-crisis levels until 2017/2018 (CSO 2018) - nearly a decade after the crisis began. This boom-bust-recovery cycle has important implications for public perception of immigration.

In the pre-crisis period, rapid immigration was often coupled with low unemployment and economic growth (Turner and Cross 2015). Unemployment fell from 16 percent in the mid-1990s to 4 percent by the turn of the next century (Ó Riain 2014). Ireland, which has a long history of emigration, found itself becoming a new context of reception in this favourable economic climate (Sheridan 2018; McGinnity and Kingston 2017) as net migration increased steadily to over 100k annually at its peak in 2007 (Fig. 1). During this period of growth, the Irish public broadly offered a warm reception to newcomers (Turner and Cross 2015) — at least to the extent that openly expressed views capture relevant sentiment in terms of the immigrant experience (McGinnity et al. 2020). McGinnity and Kingston (2017) find that attitudes to immigrants in Ireland became more negative as unemployment rose, which was moderated with greater social interaction. Once a more varied view of the relationship between immigration and the labour market is taken into account (Dancygier and Donnelly 2013), evidence suggests that economic concerns often provide a rationale for the expression of intolerance, a rationale which avoids referencing the social and cultural sources of antipathy and the associated social stigma (Creighton et al. 2015).^1^

**Fig. 1:**
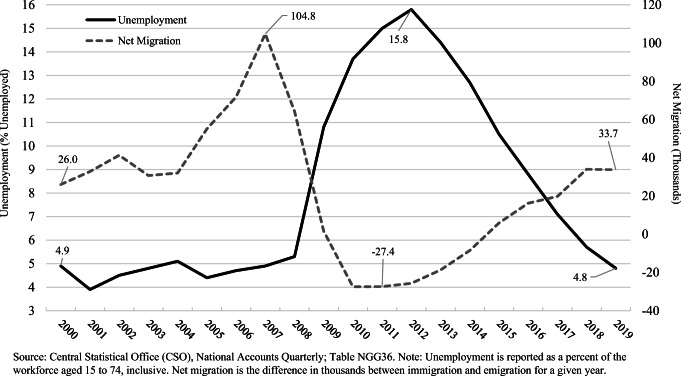
Trend in unemployment and net migration in Ireland: 2000–2019. Source: Central Statistical Office (CSO), National Accounts Quarterly; Table NGG36. Note: Unemployment is reported as a percent of the workforce aged 15 to 74, inclusive. Net migration is the difference in thousands between immigration and emigration for a given year

In the case of Ireland, economic recovery started around 2014/2015 when unemployment, which was then around 12 percent and had peaked in 2012/13 at 15.8 percent, started a sustained decline (Fig. 1). In 2015, Ireland also returned to net-positive international migration (Fig. 1). Unemployment reached pre-crisis levels of between 4 and 5 percent by 2018 (CSO 2018) and by 2019 matched that of 2000 (see Fig. 1). Although the pre-crisis and austerity periods have enjoyed significant academic scrutiny, the post-austerity period has received relatively limited attention in Europe. One reason is that many of the hardest hit economies only recently returned to pre-crisis levels on some economic measures.^2^ The implications of this new and potentially short-lived economic upturn for the perception of immigration remain largely unknown. Three options present themselves.

*One option* is where an economic downturn results in an enduring increase in anti-immigrant sentiment. In this scenario, short-term changes in the economy do not translate automatically into substantive or significant attitudinal shifts. A *second option* is that the salience of immigration fades with a decline in economic hardship, which is reflected moderated attitudes. This would suggest that the downturns in economic conditions, even as severe as that experienced during the austerity period, are ephemeral and public opinion closely tracks the economic cycle. Of course, a *third option* is a mixture of the first two. On the one hand, some characteristics of immigrants and immigration could remain a concern while others track more closely periods of boom and bust. Ireland offers a unique opportunity to assess the patterning of anti-immigrant sentiment as the economic crisis was rapid, austerity was biting and the recovery was substantive (i.e. unemployment returned to levels that closely matched the pre-crisis period).

The rest of the paper is structured as follows: first, relevant literature is reviewed; second, data and measures used are presented; third, results and their implications are discussed, and limitation outlined.

## Ethnic Competition and Implications for Anti-immigrant Sentiment

Ethnic competition^3^ theory has dominated much of the empirical work on attitudes towards immigrants (Rydgren and Tyrberg 2020; Billiet et al. 2014; Lancee and Pardos-Prado 2013; Ceobanu and Escandell 2010; Meuleman et al. 2009; Gijsberts et al. 2004). According to this framework, ethnic groups compete for scarce resources such as tangible economic goods, for example jobs and housing (Quillian 1995), but also symbolic, immaterial assets such as political power and cultural values (McLaren and Johnson 2007). Social identity theory and intergroup bias further extend this framework as individuals are more likely to perceive members of their own group (i.e., in-group) more favourably than members of other groups (i.e., outgroup) (Tajfel and Turner 1979). As a result of intergroup bias, there may be a preference to allocate scarce resources, such as jobs, to members of an in-group (Hewstone et al. 2002).

Empirical work on anti-immigration attitudes has emphasized the role of contextual factors at country level, such as economic conditions, as drivers of public sentiment (see Ceobanu and Escandell 2010 for an overview). Economic concerns linked to rate of unemployment and poor economic conditions have been associated with anti-immigration sentiment (Mayda 2006; Quillian 1995). During periods of economic upheaval and uncertainty, attitudes towards ethnic minorities are likely to become less positive due to perceived economic threat and intensified competition within the labour market. For example, studies have shown that during the economic crisis of 2007/2008 anti-immigration sentiment increased in Europe (Polavieja 2016; Billiet et al. 2014) and these negative public attitudes were linked to labour market competition. In particular, the increase in anti-immigration sentiment was noted in countries^4^ more severely impacted by economic recession (Billiet et al. 2014; Hatton 2014).

As discussed in the introduction, Ireland experienced one of the worst economic downturns in the European Union during the period of economic recession. Research in the Irish context indicates that attitudes towards immigrants are closely associated with economic conditions. During the period of economic boom, Irish attitudes were amongst the most positive in Europe (Turner 2010). However, with the onset of the recession there was a marked shift towards more negative attitudes (McGinnity and Kingston 2017; Turner and Cross 2015). As the Irish economy began to recover, attitudes towards immigrants became more favourable once again, but the welcoming public sentiment of the Celtic Tiger period did not re-emerge (McGinnity et al. 2018). This paper further builds on existing literature by examining the extent to which short-term economic recovery is matched by a shift towards more moderate attitudes towards immigration.

Individual-level characteristics such as employment status, educational attainment and general socio-economic position have also been identified as determinants of public attitudes towards immigrants (Coenders et al. 2008; Hainmueller and Hiscox 2007; Kunovich 2004). Individuals are likely to be more impacted by their own position in the labour market than the overall economic conditions and thus may express antipathy if they perceive the presence of immigrants as a threat. For example, job insecurity has been linked to greater opposition towards ethnic minorities (Kunovich 2017; Billiet et al. 2014) while employment with greater security and less exposure to competition is associated with pro-immigration attitudes (Ortega and Polavieja 2012). Similar findings can be observed in the Irish context, as individuals employed in occupations and sectors with greater job security^5^ are more likely to be supportive of further immigration while job losses, particularly in the short-term, are more likely to lead to anti-immigration sentiment (Gusciute et al. 2021). This paper further investigates Irish attitudes towards immigrants by considering the importance of education and skills in determination of attitudes.

Individuals are likely to make distinctions between different immigrant groups^6^ and may place greater importance on certain attributes over others. For example, several studies have shown that highly skilled immigrants are preferred over other immigrant groups (Helbling and Kriesi 2014; Hainmueller and Hiscox 2007; but see Helbling 2011). Education levels, which are often assumed to approximate skill levels, have linked higher levels of attained education with pro-immigration attitudes (d’Hombres and Nunziata 2016; Mayda 2006; Scheve and Slaughter 2001). While many studies have established a strong positive association between education and pro-immigration attitudes (see Ceobanu and Escandell 2010), the relative importance of education and skills in determining attitudes towards immigrants has not been considered. This is of particular importance in the Irish and European contexts as distinctions are made between skill and labour shortages, particularly in the case of non-EU immigrants. In Ireland, education and skills levels are closely aligned with the labour market and labour migration. On average, migrants in Ireland are highly educated and have a higher educational attainment than the Irish-born population; however, differences can be observed among different migrant groups in regards to occupational attainment (McGinnity et al. 2020). As these two attributes are closely linked to the labour market, it is likely they may play a significant, but distinct, role in driving pro- or anti-immigration sentiment, particularly in the context of recent economic recovery.

## Data and Analytic Sample

Table 1 reports the sample characteristics of three rounds of the European Social Survey (ESS),^7^ which each pertain to a year — 2002, 2014 and 2018 (ESS 2002, 2014, 2018). These years are selected for two reasons. First, they reflect the pre-crisis [2002], late-austerity [2014]^8^ and recovery [2018] periods in Ireland. Second, these rounds include unique measures of the perception of the importance of two immigrant attributes — education and skills — that are not part of the core questionnaire, creating a unique time-series to assess the recovery period (i.e. post-2014/2015). Moreover, this time-series is exclusive to Ireland as the questions on education and skills (see below) were only included at the country-level in 2018.^9^

**Table 1:** Descriptive statistics

	Percentage or mean (std. dev.)	*n*
Perception of importance of immigrant…		
…education (0[Ext. unimportant … 10[Ext. important])	6.51 (2.45)	6073
…skills (0[Ext. unimportant … 10[Ext. important])	7.03 (2.31)	6073
Year		
2002	30.36	1844
2014	35.83	2176
2018	33.81	2053
Age	49.27 (17.70)	6073
Sex (proportion female)	53.25	3234
Country of birth (proportion born in Ireland)	87.75	5329
Perception of education (0[Ext. bad] … 10[Ext. good])	6.48 (2.12)	6073
Education (respondent)		
Primary or less	15.94	968
Lower secondary/vocational	21.18	1286
Leaving certificate or equivalent	22.00	1336
Professional cert./apprenticeship/higher cert.	20.78	1262
Tertiary	7.81	474
Postgraduate	12.30	747
Perception of HH income		
Living comfortably	34.83	2115
Coping	45.94	2790
Difficult	14.54	883
Very difficult	4.69	285
*N*	6073	

Source: ESS 2002 [Round 1], ESS 2014 [Round 7], ESS 2018 [Round 9; Country-specific module for Ireland]

Note: Presented distribution is based on unweighted samples

As a result, understanding the change in the recovery period to public perception of the relative importance placed on skills and education is only feasible in the Irish context. ESS is also the highest quality data source on attitudes to immigrants in Ireland.^10^ Of the 6652 respondents from the pooled sample of the three rounds, 131 were missing data on one of the two outcomes, constituting about 2 percent of the initial sample. An additional 448 respondents — 7 percent of the remaining sample — were missing values on one or more of the controls used in the model (see Table 1) and were not included in the analysis. The final analytic sample includes 6073 respondents of which a third pertained to each included round of the ESS (see Table 1).

## Capturing Austerity: the Importance of Immigrant Skills and Education in Ireland

The variable that delineates the period is the year of the survey, which correspond to (1) pre-crisis [2002], (2) late-austerity [2014] and (3) recovery [2018]. The two outcomes of interest — importance of immigrant skills and education — are derived from the following questions:

*Please tell me how important you think each of these things should be in deciding whether someone born, brought up and living outside Ireland should be able to come and live here. Firstly, how important should it be for them to…*

… have good educational qualifications?

… have work skills that Ireland needs?

Respondents offer their opinion based on an 11-point scale with 0 being “extremely unimportant” and 10 being “extremely important”. These measures are admittedly limited, but they reflect a pragmatic effort to offer insight into attitudes that are plausibly different in their relationship to the labour market and are captured at key moments in the Irish economic cycle. Perception of the importance of work skills is interpreted as being closely linked to the needs of the labour market in Ireland. Educational qualifications plausibly overlap with other aspects of immigrant identity and, as such, are somewhat broader in their interpretation as the instrumental value to the labour market is less explicit. Moreover, the education question centres on the immigrant while the skills question refers to the needs of Ireland. Admittedly, these distinctions are somewhat open to interpretation, but the results indicate that the way in which they are interpreted is meaningfully distinct. Additional controls are considered that capture respondents’ demographic (age, sex, country-of-birth) and socioeconomic (education, perception of education and perception of household income) attributes (see Table 1).

## Analysis of the Perception of the Importance of Migrant Education and Skills in Ireland

**Fig. 2:**
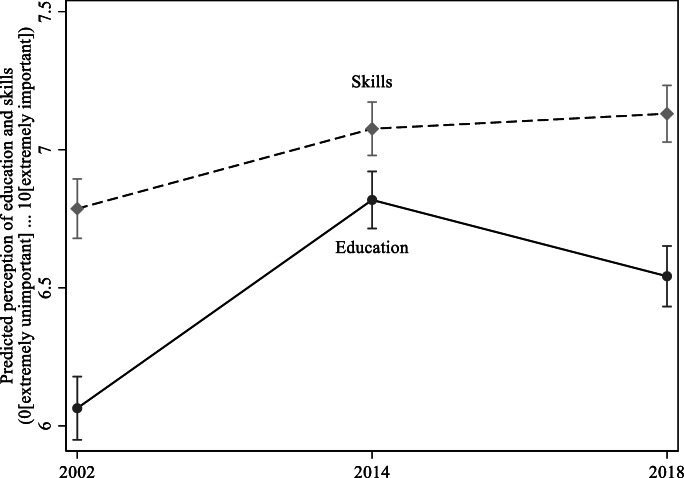
Predicted perception of importance of immigrant education and skills: Ireland 2002, 2014 and 2018. Source: ESS 2002 [Round 1], ESS 2014 [Round 7], ESS 2018 [Round 9; country-specific module for Ireland]. Notes: The reported values for a given year based on coefficients estimated on the full model (columns 3 and 6; Table 2). For each year, all other measures included in the model are held at their means

Table 2 reports the coefficients and relevant tests of significance for a linear regression of the perception of the importance of education or skills. The six models, three for each outcome, reflect incremental additions of demographic and socioeconomic controls. The models are intended to be descriptive in their interpretation. Figure 2, which is based on the model in Table 2 with the full set of covariates (i.e. column 3 and 6), visually shows the estimated mean importance placed on education/skills and the associated 95 percent confidence intervals. Three notable patterns emerge.

**Table 2:** Linear regression of the perception of the importance of immigrant education and skills on demographic and socioeconomic attributes of the respondent: Ireland 2002, 2014 and 2018

	Perception of Education [0–10]†			Perception of Skills [0–10]†		
	*b* (std. err.)			*b* (std. err.)		
Year (ref. = 2018)						
2002	−0.507***	−0.426***	−0.497***	−0.385***	−0.287***	−0.341***
	(0.08)	(0.08)	(0.08)	(0.07)	(0.08)	(0.08)
2014	0.238**	0.287***	0.253***	−0.083	−0.022	−0.051
	(0.08)	(0.08)	(0.08)	(0.07)	(0.07)	(0.07)
Age		−0.024*	−0.016		0.008	0.015
		(0.01)	(0.01)		(0.01)	(0.01)
Age squared		0.000***	0.000*		0.000	−0.000
		(0.00)	(0.00)		(0.00)	(0.00)
Sex (ref. = male)		−0.037	−0.032		−0.164**	−0.162**
		(0.06)	(0.06)		(0.06)	(0.06)
Country of birth (ref. = Ireland)		0.272**	0.295**		0.052	0.070
		(0.10)	(0.10)		(0.09)	(0.09)
Perception of education			0.104***			0.085***
(0[Ext. bad] … 10[Ext. good])			(0.01)			(0.01)
Education (ref. = primary or less)						
Lower secondary/vocational			−0.013			−0.161
			(0.11)			(0.11)
Leaving certificate or equivalent			−0.189			−0.204
			(0.11)			(0.11)
Prof. cert./Apprent./Higher cert.			−0.203			−0.277*
			(0.12)			(0.11)
Tertiary			−0.430**			−0.222
			(0.15)			(0.14)
Postgraduate			−0.511***			−0.483***
			(0.14)			(0.13)
Perception of HH income (ref. = Living comf.)						
Coping			0.039			−0.030
			(0.07)			(0.07)
Difficult			0.144			0.164
			(0.10)			(0.10)
Very difficult			0.213			0.334*
			(0.16)			(0.15)
Intercept	6.564***	6.762***	6.177***	7.153***	6.568***	6.180***
	(0.05)	(0.22)	(0.26)	(0.05)	(0.21)	(0.25)
R-squared	0.015	0.022	0.032	0.005	0.021	0.029
*N*	6073	6073	6073	6073	6073	6073

*p < 0.05, **p < 0.01, ***p < 0.001

Source: ESS 2002 [Round 1], ESS 2014 [Round 7], ESS 2018 [Round 9; Country-specific module for Ireland]

Note: Regression models use sample weights

†The outcome of interest is an 11-point scale with 0 indicating the outcome is “extremely unimportant” and 10 indicating the outcome is “extremely important”

*First*, work skills relative to educational qualification are perceived to be more important by the public in Ireland regardless of the period of observation (see Fig. 2). The gap is particularly pronounced in the pre-crisis period [2002] with some indication of a narrowing during late-austerity [2014]. The estimates from the recovery period [2018] signal a widening once again, but the gap is less pronounced. The implication is that periods of economic crisis plausibly result in less distinction between skills and education, marked by a reduced gap between the two measures, but the greater importance placed on skills relative to education is a somewhat stable pattern that does not disappear or flip for the periods observed.

*Second*, the results confirm that immigrants confronted a less receptive landscape towards the end of austerity. The estimated coefficient for period/year, which offers one view of the difference in the outcome of interest for a given year relative to the recovery period (i.e. 2018), is meaningfully different for the late-austerity period (i.e. 2014) regardless of whether skills or education is being considered. The general trend is a substantive increase in the importance placed on immigrant education and skills. In addition, this change between the pre-crisis and late-austerity period remains in a model that controls for demographic and socioeconomic attributes of the individual, suggesting that the economic period is a strong predictor.

*Third*, Fig. 2 highlights the distinct trajectories of attitudes towards skills and education. When considering the former, evidence suggests that an improvement in the economic context does not diminish the perceived importance of immigrants offering a skillset that Ireland “needs”. In other words, although the economic context changed and with plausibly distinct skill needs emerging, the hardening of sentiment towards immigrants on the issue of skills in terms of public perception, which increased when austerity arrived, remains unchanged in the recovery period. In contrast, there is a decline in the perception that education is important for immigrants although there is no return to the pre-crisis levels. In fact, 2002 and 2014 are both higher and lower than 2018, respectively. This distinct trajectory is reflected Table 2 as 2014 [late-austerity] is positively different than 2018 [recovery] only in the model considering educational qualifications of the immigrant. Put another way, the only substantive decline in public perception of the importance of any immigrant attribute is for education between 2014 and 2018.

## Implications

These results suggest that that the public raises the bar when evaluating immigrant attributes in times of austerity. Specifically, the skills and educational qualifications become more salient after the economic crisis of 2007/2008 and at the height of austerity. This observation, evidenced in the results, is largely confirmational and is unlikely to be context-specific to Ireland. However, the observed pattern does not sustain the broader claim that public sentiment tracks the economy for better or worse. In particular, the greater importance of work skills relative to educational qualification points to concern about the needs of the labour market and thus a preference for immigrants who can meet those needs. Although the downturn paralleled an increase in rigidity of attitudes towards immigration, the recovery did not usher in an unambiguous return to pre-crisis levels of receptivity.

We suggest that these results, albeit limited in terms of scope and measures, indicate that the timing of recovery in public opinion should not be expected to track the economic cycle — at least in the short-term. In the Irish case, public perception that immigration needs to be of material benefit to the Irish economy remained consistent in 2018 relative to 2014. This stability is despite a notable change in economic circumstances at least at the macro-level, which suggests that macro-economic patterns are not always indicative of the trajectory of public opinion. However, the limited evidence presented here cautions against over-generalization. Because the apparent value of educational qualifications declines in the eyes of the public in post-austerity Ireland, we suggest that the importance placed on attributes of the immigrant that are less tightly linked to the labour market might be more synchronized with the broader economic outlook.

The implications for our broader understanding of public perception of immigration and economic context are clear. The economic climate tracks public opinion, but only in a conditional sense. We reveal that scarring — a process by which the experience of economic precarity in the recent past shapes current public perception — meaningfully defines society’s reception of immigrants. It may well be the case that longer-term, stable economic upturns eventually translate into something similar to that observed in the pre-crisis period. But, an economic recovery clearly does not moderate opinion as rapidly as a crisis erodes it. Moreover, the context of reception in austerity-hit economies retains a less moderate view when it comes to skills. This targeted perception highlights the way that immigrants are interpreted as needing to be justified by explicit labour-market need, but more indirectly applicable attributes (e.g. education) experience a more rapid moderation in antipathy. In short, the scars are deep and lasting, but some do heal.

## Limitations and Future Work

The research paper is insightful in that it highlights changes in the perception of immigration during short-term economic recovery in a country severely impacted by the economic crisis of 2007/2008. However, three issues deserve mention. First, the attitudinal measures are limited in scope. The difference in the boom-bust-recovery trend in attitudes towards immigrant education and skills indicates that the two measures capture substantively different attributes. However, some sociocultural dimensions of anti-immigrant sentiment remain unaddressed. As both skills and, to a lesser extent, education capture human capital dimensions of immigrant economic incorporation (Mayda 2006), it would be of interest to see if cultural concerns (e.g. religion, national identity, race/ethnicity) are equally sensitive to economic fluctuations. An international comparative perspective, assuming comparable measures are available, would improve the generalizability of the conclusions.

The second limitation is the reliance on one dimension of the expression of sentiment directed at immigrants. Recent work, including some in Ireland, has shown that attitudes towards immigrants can be significantly and substantively masked (Creighton et al. 2015; McGinnity et al. 2020; Creighton et al. 2022). The ESS, which is a door-to-door, household survey is ill equipped to address biases in reporting due to respondents seeking to leave a more socially desirable impression. In the US, this bias has been shown to explain variation in pre- and post-crisis support for a closed border (Creighton et al. 2015). As a result, the work here should only be interpreted as capturing openly expressed attitudes towards immigrants/immigration, which is informative but limited.

Third, there are notable concerns linked to anti-immigrant sentiment that are independent of or only indirectly related to the labour market. These are not excluded as relevant to the Irish context in the analysis presented here but should be explored in greater detail. That is not to say that these perspectives are absent from the literature in Ireland. Recent work in Ireland by Creighton et al. (2022) considered the role of race and religion in masked intolerance. Loyal and Quilley (2016, 2018) highlighted he role of the state in asylum-based regimes. Fanning (2018) considered the increasingly diverse migrant community. Joseph (2018) explored how migrants to Ireland understand and confront the existing racial hierarchy. That said, future work would do well to explore how non-materialist concerns can be key mechanism in the formation of attitudes towards newcomers.

Overall, this analysis would benefit from more frequent measurement of public sentiment as the time period between 2002 and 2014 is notably large. In the end, we consider this work to be an important, albeit modest and descriptive, step towards a clearer understanding of the short-term implications of economic crisis and recovery for public perception of immigrants/immigration. We focused on two dimensions that are, to differing extents, linked to the labour-market, but in plausibly distinct ways. Results suggest that even with this limited set of measures, notable differences emerged in the trajectory of public perception in the recovery period. This suggests that the implications of the economic crisis for newcomers to Ireland — and perhaps elsewhere — will be felt for some time even if the initial economic circumstances are somewhat less relevant.

## Footnotes

1 In the Irish case, immigration did not play a large role in national politics (O’Malley 2008), indicating that increasing anti-immigrant sentiment in terms of public opinion did not translate into a constituency.

2 The economic effects of the public health response to COVID-19 suggest that this window of recovery might be very short-lived indeed as unemployment has increased dramatically since the start of the pandemic in 2020 having only returned to the level of 2000 in 2019 (see Fig. 1).

3 Also referred to as competition theory and group conflict theory.

4 Hatton (2014) notes a more pronounced increase in anti-immigration sentiment in Spain, Greece and Ireland in comparison to other European countries.

5 Job growth and job losses are used to measure job security. See Gusciute et al. 2021.

6 Note that ethnic hierarchy is outside of the scope of this paper. See Ford 2011; Hagendoorn 1993.

7 The European Social Survey (ESS) in Ireland is publicly available and accessed via a registration process at the following URL: https://www.europeansocialsurvey.org/. For ESS Round 9, the interview and sampling protocols were fully reviewed and approved by the Office of Research Ethics at University College Dublin where the fieldwork was coordinated. In all rounds, informed consent was a requirement for participation.

8 As shown in Fig. 1, unemployment peaked in 2012/2013. Given available data, we consider the lag in measurement of public opinion to be reasonable as unemployment remained nearly 3 times that of the pre-crisis period when ESS exited the field in 2014. It was the 2015 budget that was considered the first to relax, albeit slightly, austerity measures (RTÉ 2014). Also, it was not until 2015 that Ireland experienced a return net positive international migration (Bergin et al. 2020).

9 There are a number of questions included in each round of the ESS that are implemented by the national coordination for a single country. In 2018, Ireland repeated the 2002 and 2014 measures on the importance of skills and education for immigration, which created a unique time-series shared by no other country participating in the ESS.

10 There is a notable absence of ongoing surveys of attitudes to immigrants and immigration in Ireland (Fahey et al. 2019), underlining the unique insight of the ESS-derived time series.
